# Evaluating the Evolving Real‐World Adverse Events of GLP‐1RAs Using FDA Adverse Event Reporting System (FAERS)

**DOI:** 10.1002/oby.70176

**Published:** 2026-03-09

**Authors:** David Stone, Mary Playdon, Stephen D. Hursting, Aik Choon Tan

**Affiliations:** ^1^ Departments of Oncological Sciences and Biomedical Informatics University of Utah Salt Lake City Utah USA; ^2^ Huntsman Cancer Institute University of Utah Salt Lake City Utah USA; ^3^ Department of Nutrition and Integrative Physiology University of Utah Salt Lake City Utah USA; ^4^ Department of Nutrition University of North Carolina at Chapel Hill Chapel Hill North Carolina USA; ^5^ Nutrition Research Institute University of Carolina at Chapel Hill Kannapolis North Carolina USA; ^6^ Lineberger Comprehensive Cancer Center University of North Carolina at Chapel Hill Chapel Hill North Carolina USA

## Abstract

**Objective:**

This study aimed to assess the spectrum and frequency of adverse events (AEs) linked to glucagon‐like peptide‐1 receptor agonists (GLP‐1RAs) using the US FDA Adverse Event Reporting System (FAERS). Emphasis was placed on emerging safety concerns in context‐specific use.

**Methods:**

A retrospective analysis of FAERS reports between 2012 and 2025 was conducted. Five commonly prescribed FDA‐approved GLP‐1RAs were included. Disproportionality analyses were applied to detect AE signals. Subgroup analyses evaluated associations by indication, GLP‐1RAs compared to other drugs, and AEs specific to individual GLP‐1RAs.

**Results:**

From over 18 million FAERS reports, 137,451 involved GLP‐1RAs. The most frequent AEs were gastrointestinal, nutritional and metabolic, and psychiatric disorders, occurring at higher rates compared to other drugs. In diabetes use, GLP‐1RAs were associated with retinopathy, hearing loss, and cataracts. In contrast, when prescribed for weight management/obesity, nutritional, metabolic, and psychiatric AEs predominated. We also developed an open‐access portal for AE exploration, available at http://glp1.tanlab.org.

**Conclusions:**

GLP‐1RAs are linked to a broad range of AEs across indications. These findings stress the need for careful clinical monitoring and long‐term safety evaluation. This study also illustrates how real‐world evidence can inform safety communications, as well as hypothesis generation for research on next‐generation GLP‐1RAs.

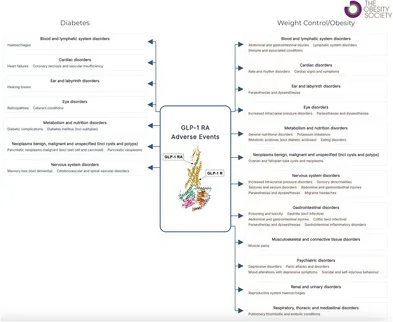

## Graphical abstract

Web portal for interrogating Adverse Events of GLP‐1 RA in diabetes vs weight control/obesity.

## 1 Introduction

Obesity is one of the most pressing public health challenges of the 21st century. The prevalence of overweight and obesity has risen steadily over the past four decades, with the World Health Organization estimating that over 890 million adults worldwide are currently living with obesity [1]. The obesity epidemic contributes to an enormous burden of chronic disease, including type 2 diabetes (T2D), cardiovascular disease, and several cancers, as well as psychosocial and economic consequences. Despite widespread recognition of the epidemic's impact, sustainable and effective treatments for obesity and weight control have historically been limited.

In recent years, glucagon‐like peptide‐1 receptor agonists (GLP‐1RAs) have emerged as transformative pharmacological options for obesity and weight control management. Originally developed for the treatment of T2D, GLP‐1RAs mimic the endogenous incretin hormone GLP‐1, stimulating insulin secretion, inhibiting glucagon release, slowing gastric emptying, and promoting satiety [2, 3]. GLP‐1RAs represent a major advance in the treatment of obesity, offering unprecedented efficacy in reducing body weight and improving cardiometabolic health [4]. However, their rapid adoption underscores the urgency of understanding their full safety profile in real‐world practice.

Given the rapid uptake of GLP‐1RAs in clinical practice, post‐marketing surveillance is essential to identify and characterize safety signals not captured in trials. Regulatory authorities such as the US Food and Drug Administration (FDA) rely heavily on spontaneous reporting systems to monitor adverse drug events after approval. The FDA Adverse Event Reporting System (FAERS) is one of the largest publicly available pharmacovigilance databases, containing millions of reports submitted by health care professionals, patients, and manufacturers. Previous studies have used FAERS to investigate specific adverse events (AEs) related to GLP1‐RAs [5, 6, 7] or combinations of GLP‐1RAs with other drugs [8].

In this study, we performed a systematic analysis of AEs of GLP‐1RAs using the FAERS database. Through this analysis, we aimed to provide clinicians, researchers, and policy makers with a more comprehensive understanding of the safety profile of GLP‐1RAs in a context‐specific manner. These findings will guide patient counseling and support the development of strategies to maximize therapeutic benefit while minimizing harm.

## 2 Methods

### 2.1 FAERS Database and Cohort Selection

We downloaded FAERS quarterly‐reported AEs data, which consists of 18,499,609 cases from 2012 Q4 to 2025 Q1. As the AE cases were reported quarterly, some cases were inadvertently reported in multiple quarters, and we performed data preprocessing to remove duplicate records across quarters. We used MedDRA High Level Terminology (HLT) to harmonize the AEs data.

### 2.2 Glucagon‐Like Peptide‐1 Receptor Agonists (GLP‐1RAs)

We focused on the five commonly prescribed FDA‐approved GLP‐1RAs, which include: exenatide (FDA‐approval year 2005); liraglutide (FDA‐approval year 2010); dulaglutide (FDA‐approval year 2014); semaglutide (FDA‐approval year 2017); and tirzepatide (FDA‐approval year 2022).

### 2.3 Dipeptidyl Peptidase‐4 Inhibitors (DPP4i)

As comparator for GLP‐1RAs used in T2D treatment, we included three DPP4i: sitagliptin, saxagliptin, and linagliptin in this study.

### 2.4 Sodium‐Glucose Cotransporter 2 Inhibitors (SGLT2i)

We also used SGLT2i, another T2D treatment, as comparator for GLP‐1RAs. The three FDA‐approved SGLT2i used were canagliflozin, dapagliflozin, and empagliflozin.

### 2.5 Disproportionality Analysis

For each GLP‐1RA‐AE pair, we performed disproportionality analysis using two common methods for signal detection in pharmacovigilance data: reporting odds ratio (ROR) and proportional reporting ratio (PRR) [9, 10, 11]. These disproportionality calculations are based on the two‐by‐two contingency table for each GLP‐1RA‐AE pair. ROR focuses on the odds of AEs occurring for a given drug versus other drugs while PRR focuses on the proportion of AEs that have occurred among reports for a given drug versus other drugs, as previously described [12]. We also performed a chi‐square test on the contingency table to estimate the *p* value of a particular GLP‐1RA‐AE pair, and the Benjamini–Hochberg correction was applied to account for the multiple testing. We used the cutoff of ROR (or PRR) greater than log2 of 1 and adjusted *p* value < 0.05 for significant GLP‐1RA‐AE pairs. Results were visualized as volcano plots.

### 2.6 Computation of AEs Co‐Occurrence

To compute the co‐occurrence of AEs, we used the overlap coefficient, also known as the Szymkiewicz–Simpson coefficient, which is defined as the size of the intersection of set A and set B over the smaller set size between A and B. Assuming *X* and *Y* are two sets of cases with AE_*X*_ and AE_*Y*_, to compute the co‐occurrence of these two AEs, we calculated the overlap coefficient formula: overlap(*X*,*Y*) = |*X* ∩ *Y*|/min(|*X*|,|*Y*|). Results were visualized as heat maps.

## 3 Results

### 3.1 Cohort Description

**FIGURE 1:**
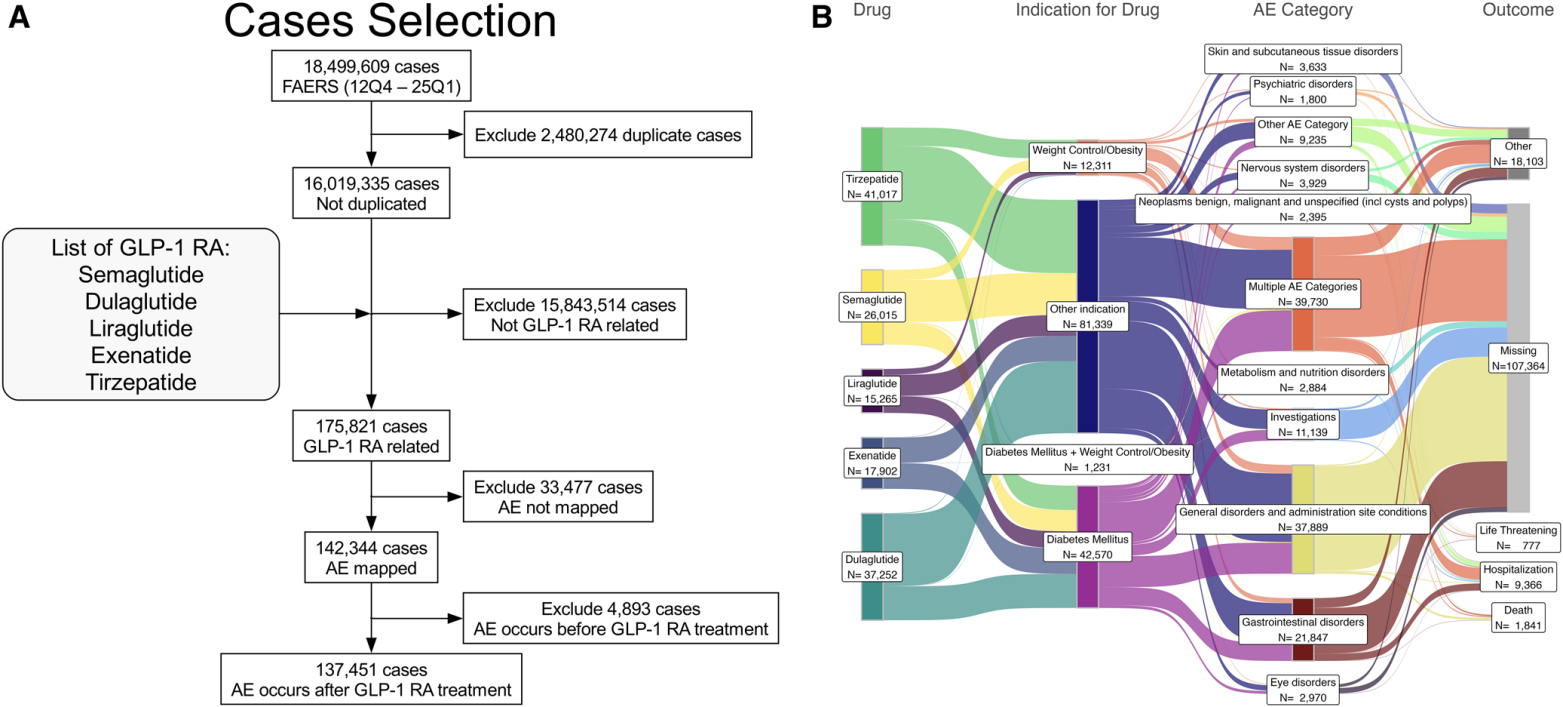
(A) CONSORT diagram of cohort selection of GLP‐1RAs and AEs from FAERS dataset. (B) Sankey plot of the five GLP‐1RAs investigated in this study, the drug indications, top 10 AE categories, and clinical outcomes.

We downloaded FAERS data from 2012 Q4 to 2025 Q1, which consists of 18,499,609 cases. After removing duplicate cases, there were 175,821 (0.95%) cases involved in the five commonly prescribed FDA‐approved GLP‐1RAs in the United States. We further excluded cases that were not mappable to an AE category (33,477 cases) and cases in which the reported AEs occurred before GLP‐1RA treatment (4893 cases) (Figure 1A).

We focused on the resultant 137,451 cases, where tirzepatide (41,017 cases, 29.8%) accounted for the largest share of AE reports, followed by dulaglutide (37,252 cases, 27.1%), semaglutide (26,015 cases, 18.9%), exenatide (17,902 cases, 13.0%), and liraglutide (15,265 cases, 11.1%) (Figure 1B).

From the 137,451 reported cases, GLP‐1RAs were used for diabetes treatment (42,570 cases), followed by weight control/obesity (12,311 cases) and both diabetes and weight control/obesity together (1231 cases). There were 81,339 cases where GLP‐1RAs were used for other treatment (off‐label usage) or indication was not clearly indicated or unavailable (Figure 1B). Serious outcomes were reported in 8.7% of cases, including hospitalization (6.8%), life‐threatening events (0.6%), and death (1.3%) (Figure 1B).

### 3.2 AEs Overview

**FIGURE 2:**
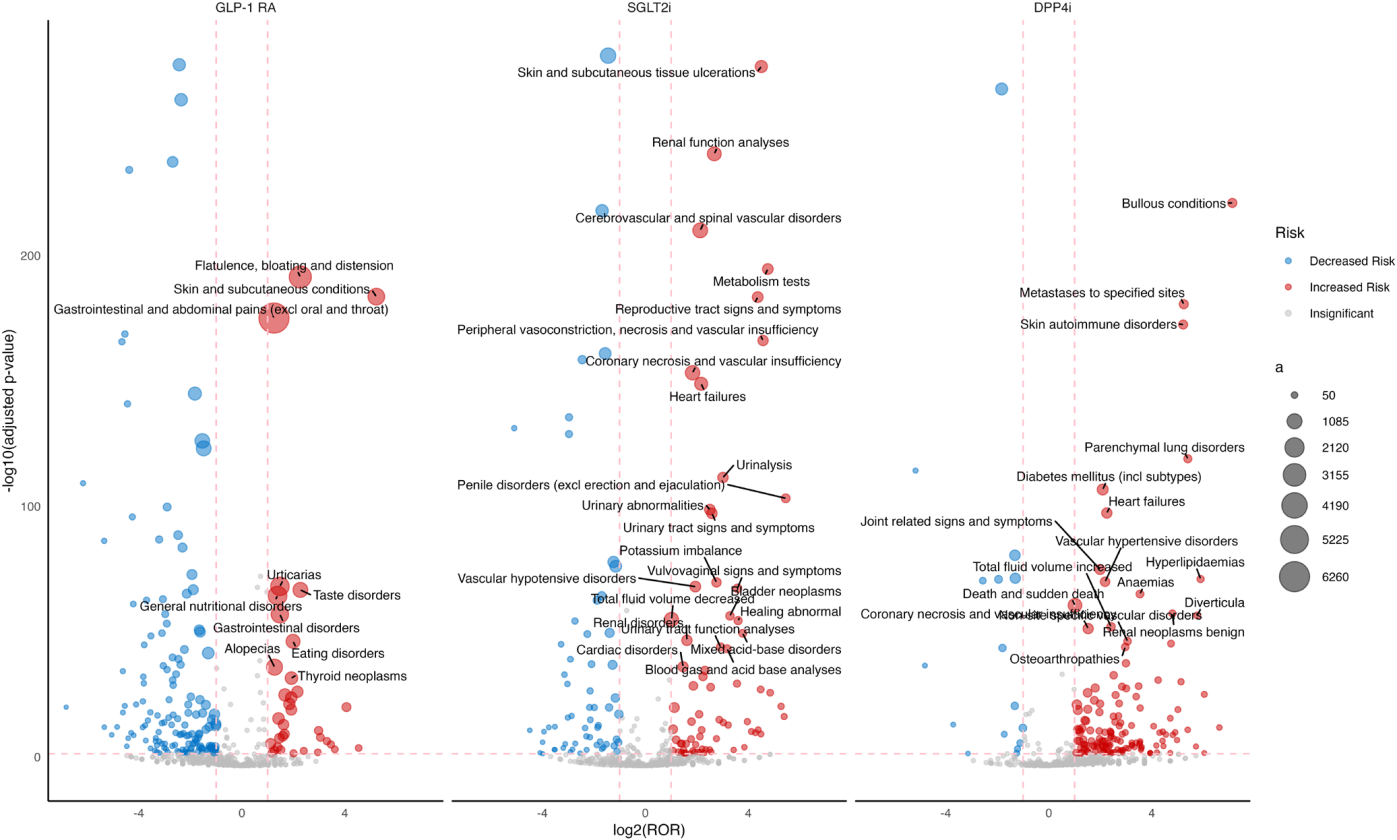
Volcano plot of the reporting odds ratio (ROR) of AEs significantly associated with GPL‐1RAs, SGLT2i, and DPP4i.

To investigate the AEs associated with GLP‐1RAs, we first compared ROR with two other commonly used T2D treatments, DPP4i and SGLT2i, in the FAERS dataset (Figure 2).

As illustrated in Figure 2, the number of AEs significantly associated with GLP‐1RAs, SGLT2i, and DPP4i were 35, 71, and 146, respectively. As compared to DPP4i and SGLT2i, GLP‐1RAs were more associated with AEs related to skin and subcutaneous conditions (ROR 37.24, 95% CI 22.75–60.95); flatulence, bloating, and distension (ROR 4.81, 95% CI 4.23–5.46); gastrointestinal and abdominal pains (excluding oral and throat) (ROR 2.37, 95% CI 2.22–2.53); alopecias (ROR 2.40, 95% CI 2.08–2.77); taste disorders (ROR 4.83, 95% CI 3.89–5.99); eating disorders (ROR 3.96, 95% CI 3.19–4.91); mood alterations with depressive symptoms (ROR 3.06, 95% CI 2.27–4.13); general nutritional disorders (ROR 2.61, 95% CI 2.32–2.94); gastrointestinal disorders (ROR 2.77, 95% CI 2.42–3.18); and suicidal and self‐injurious behaviors (ROR 2.68, 95% CI 2.11–3.40). For the general nutritional disorders, this AE category includes the following nutritional disorders: feeding disorder, food aversion, malnutrition, starvation, marasmus, and protein deficiency.

GLP‐1RA‐associated AEs were mostly related to gastrointestinal, nutritional and metabolic, and psychological disorders, as compared to DPP4i and SGLT2i.

### 3.3 AEs of GLP‐1RA Usage in Diabetes and Weight Control/Obesity

**FIGURE 3:**
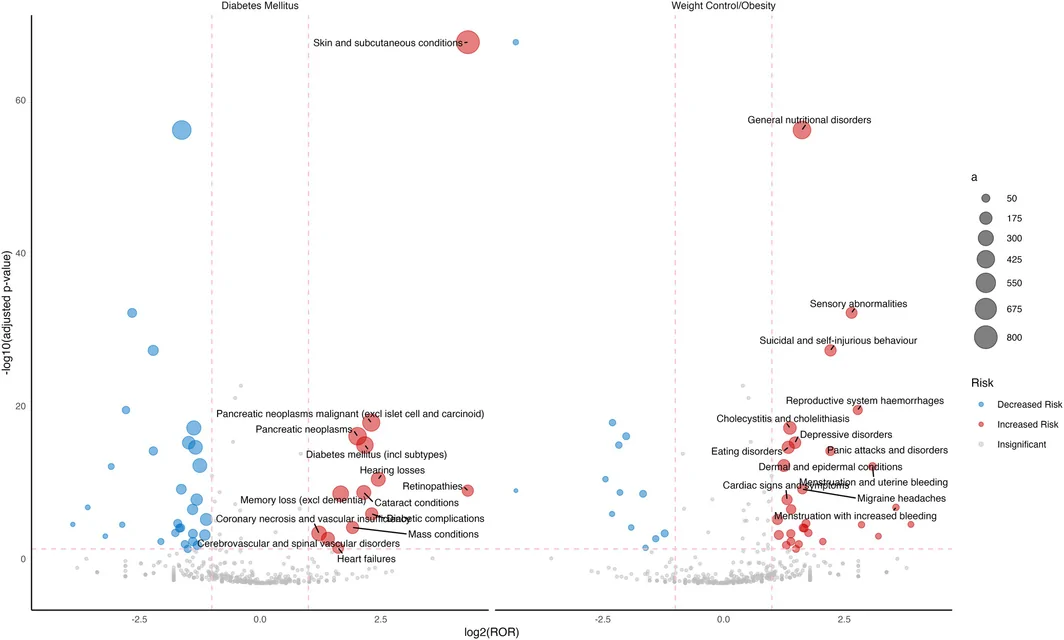
Volcano plot of the reporting odds ratio (ROR) of AEs significantly associated with GPL‐1RA usage in T2D and weight control/obesity.

Next, we investigated AEs of GLP‐1RA usage in diabetes and weight control/obesity. We performed disproportionality analysis on the two groups of GLP‐1RAs' intended usage—diabetes (T2D) and weight control/obesity (Figure 3).

From our analysis, we compared 531 GLP1‐RA‐associated types of AEs and found that 92% (489) of these AEs were similar between usage of GLP‐1RAs in diabetes treatment and weight control/obesity treatment. Furthermore, 13 (2.45%) AEs were associated with increased risk in diabetes treatment and 5.46% (29) AEs were associated with weight control/obesity usage.

GLP‐1RA usage in diabetes was associated with AEs related to retinopathies (ROR 19.73, 95% CI 4.88–79.69); skin and subcutaneous conditions (ROR 19.75, 95% CI 11.17–34.94); pancreatic neoplasms malignant (excluding islet cell and carcinoid) (ROR 4.93, 95% CI 3.26–7.44); pancreatic neoplasms (ROR 4.05, 95% CI 2.81–5.84); hearing losses (ROR 5.45, 95% CI 3.12–9.53); and cataract conditions (ROR 4.43, 95% CI 2.67–7.35) (Figure 3).

Conversely, AEs related to GLP‐1RA usage in weight control/obesity were associated with general nutritional disorders (ROR 3.08, 95% CI 2.70–3.51); panic attacks and disorders (ROR 4.63, 95% CI 3.28–6.54); sensory abnormalities (ROR 6.28, 95% CI 4.68–8.41); suicidal and self‐injurious behavior (ROR 4.64, 95% CI 3.59–6.00); immune and associated conditions (ROR 3.13, 95% CI 2.10–4.66); eating disorders (ROR 2.53, 95% CI 2.07–3.09); and depressive disorders (ROR 2.79, 95% CI 2.24–3.46). For immune and associated conditions, this includes inflammation, Crohn's disease, ulcerative colitis, immune system disorder, interstitial cystitis, and uveitis.

For female patients treated with GLP‐1RAs for weight control/obesity, there was higher reporting of AEs related to menstruation with increased bleeding (ROR 11.88 95% CI 5.12–27.57); ovarian and fallopian tube cysts and neoplasms (ROR 14.71, 95% CI 4.95–43.74); and reproductive system hemorrhages (ROR 6.86, 95% CI 4.64–10.14) (Figure 3).

### 3.4 AEs Between Different GLP‐1RAs

The five GLP‐1RAs evaluated have multifaceted treatment benefits, along with unique modes of action and toxicity profiles. As GLP‐1RAs bind to and activate the GLP‐1 receptor to exert their effects, their specific structure influences binding affinity, half‐life, and distribution. For example, exenatide was engineered with a polyethylene glycol conjugation attached to the exendin‐4 structural backbone to extend half‐life. Other GLP‐1RAs have their backbone structure modified with albumin conjugation (dulaglutide) or fatty acid chains (liraglutide, semaglutide) to extend their duration of action. In contrast, tirzepatide is a dual glucose‐dependent insulinotropic polypeptide/GLP‐1 agonist, binding to both receptors, though with weaker affinity for the GLP‐1 receptor compared to native GLP‐1. Their pharmacological activities are also influenced by their different structural backbones and pharmacokinetic properties [13, 14].

**FIGURE 4:**
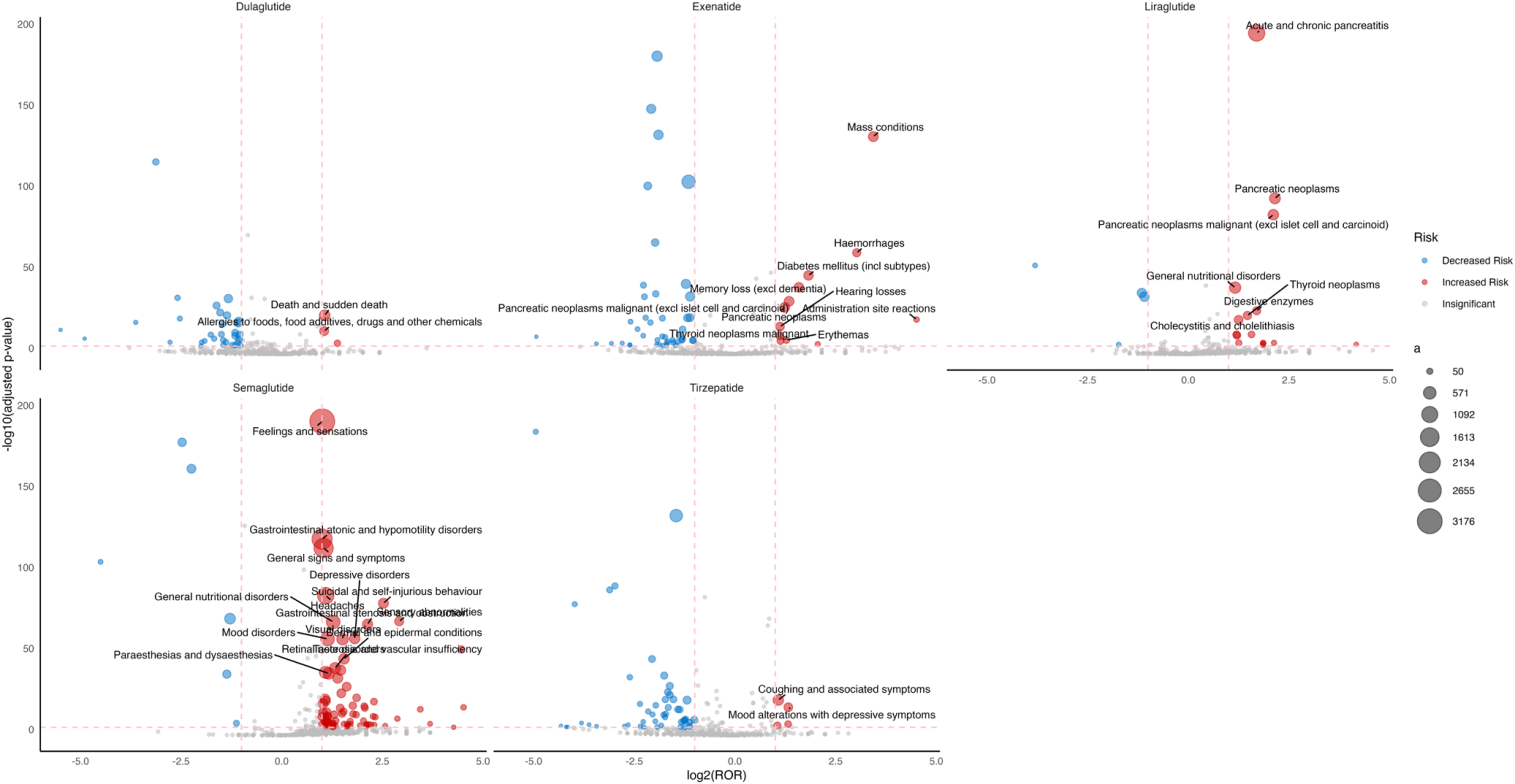
Volcano plot of the reporting odds ratio (ROR) of AEs significantly associated with seven GPL‐1RAs: dulaglutide, exenatide, liraglutide, semgalutide and tirzepatide.

To assess the toxicity profiles between the five GLP‐1RAs, we performed disproportionality analysis using ROR for each GLP‐1RA against other GLP‐1RAs (e.g., semaglutide vs. the other four GLP‐1RAs). As illustrated in Figure 4, the different GLP‐1RAs have different toxicity profiles.

**FIGURE 5:**
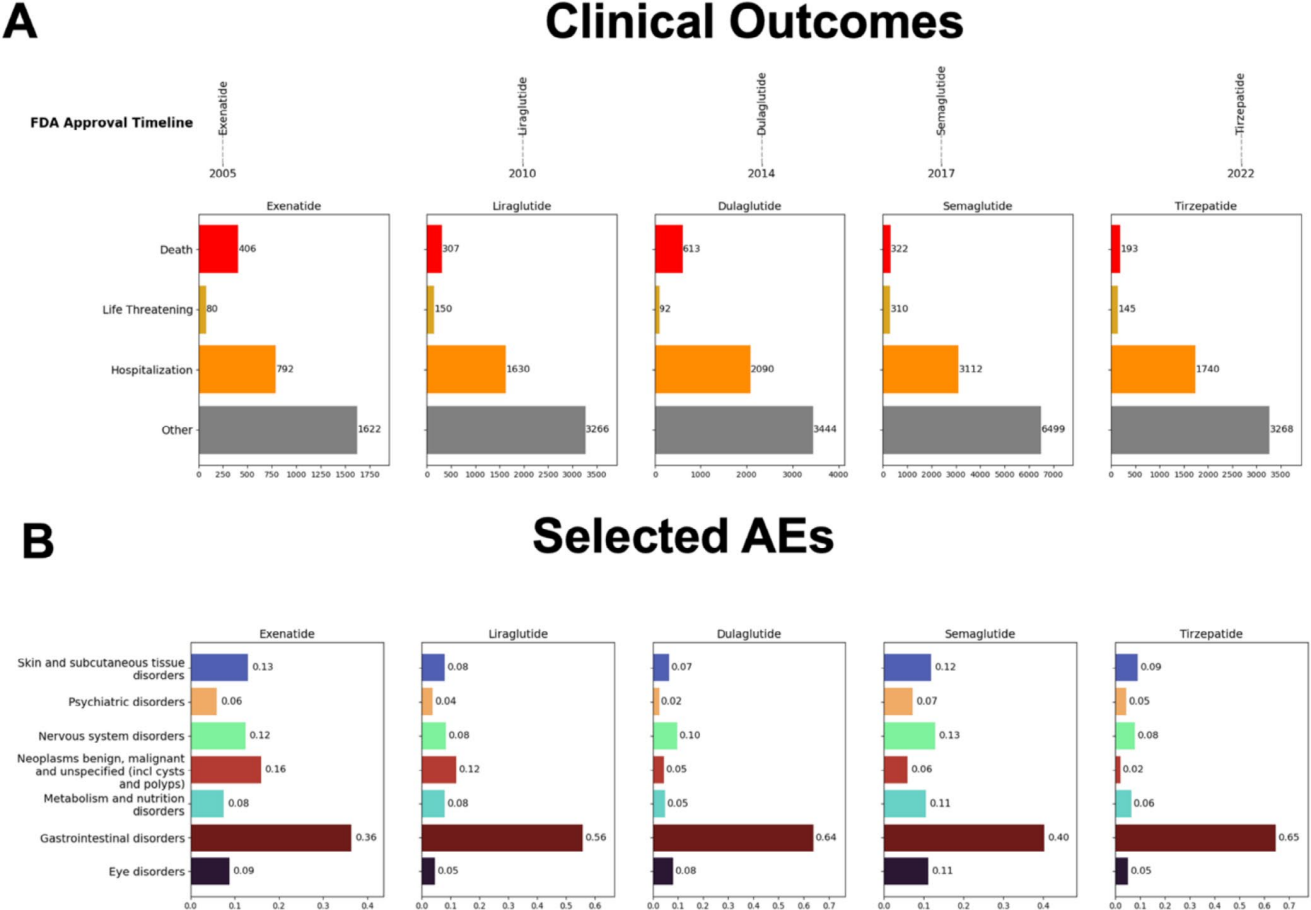
Evolving real‐world AEs of five GLP‐1RAs based on FDA‐approval year. (A) Clinical outcomes of the AEs. (B) Selected AEs associated with the seven GLP‐1RAs.

We observed different GLP‐1RAs have different toxicity profiles (Figure 4). Treatment with semaglutide was associated with an increased occurrence of gastrointestinal side effects, psychiatric disorders, and metabolism and nutritional disorders (Figure 4). Dulaglutide and exenatide were associated with more deaths and hospitalization events, and liraglutide and exenatide had a higher proportion of AEs associated with neoplasms (Figures 4 and 5A). In general, the newer GLP‐1RAs were associated with a lower occurrence of fatal or life‐threatening AEs (Figure 5A), but more related to psychiatric disorders, relative to early generation GLP‐1RAs. Gastrointestinal disorders were prevalent with all GLP‐1RAs (Figure 5B). This highlights the evolving real‐world AE profiles associated with GLP‐1RAs.

### 3.5 Co‐Occurrence of AEs

**FIGURE 6:**
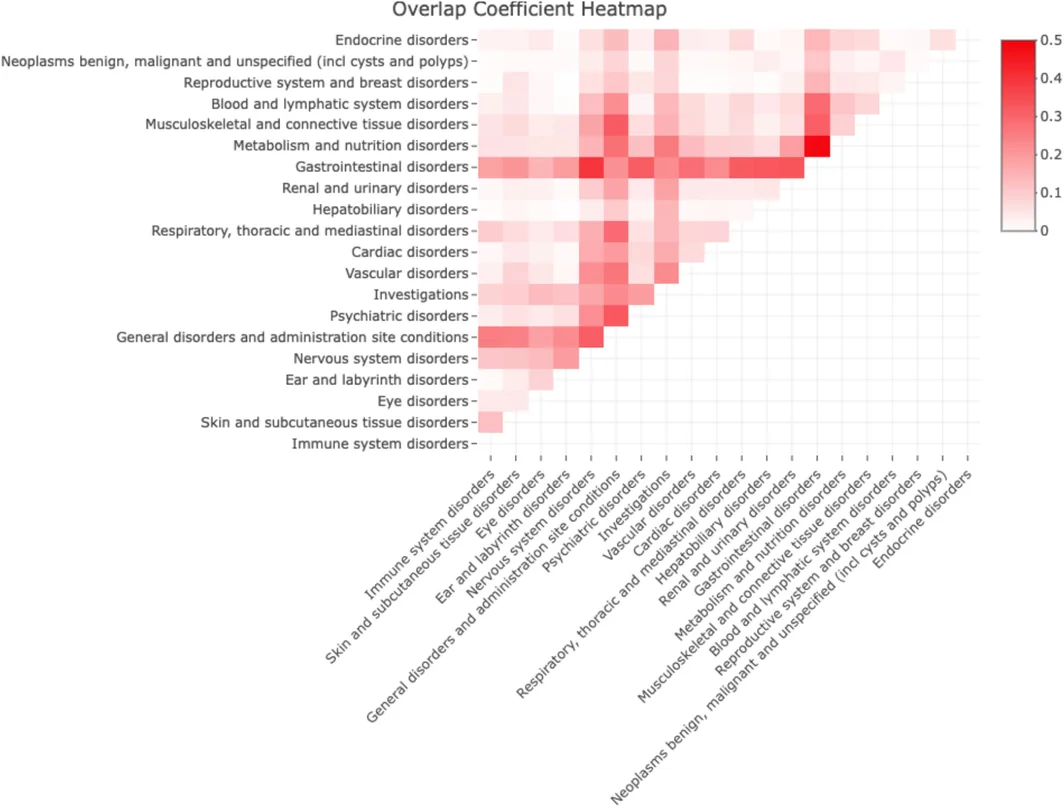
Co‐occurrence of AEsassociated GLP‐1RAs.

We also investigated the co‐occurrence of AEs for patients treated with GLP‐1RAs. We computed overlapping coefficients for all pairwise AEs at the System Organ Classes (SOCs) and plotted the co‐occurrence of AEs (Figure 6). Several AEs, such as metabolism and nutrition disorders co‐occurred with gastrointestinal disorders; Nervous system disorders co‐occurred with gastrointestinal disorders, and some AEs co‐occurred more frequently than other AEs.

### 3.6 Web Portal to Explore AEs of GLP‐1RAs

Finally, we have developed a user‐friendly web portal for users to explore additional AEs associated with GLP‐1RAs. The web portal is freely available at http://glp1.tanlab.org. All the results presented in this study are available in the web portal. In addition, PRR results were also presented in the web portal. This web portal will allow users to perform additional analyses and visualizations of the AEs associated with GLP‐1RAs. We believe that this web portal will be a useful resource for researchers to generate new hypotheses of GLP‐1RAs' toxicities, as well as identify new and unexpected AEs related to GLP‐1RAs.

## 4 Discussion

In this study, we performed a systematic analysis of FAERS, a large‐scale pharmacovigilance database, in evaluating the evolving toxicity profiles of GLP‐1RAs. FAERS provides unparalleled real‐world information on the adverse effects of GLP‐1RAs across diabetes treatment, weight control, obesity, and other usage. Using disproportionality analyses such as ROR, we compared GLP‐1RAs against other diabetes drugs (DPP4i and SGLT2i) for surveying the AEs associated with each drug. In addition, we compared the usage of GLP‐1RAs in diabetes and weight control/obesity treatments to uncover different toxicity profiles in different usages. We also evaluated the AEs associated with individual GLP‐1RAs, identifying toxicity profiles associated with different GLP‐1 treatments, potentially due to the backbone structure of the drugs. We also studied the co‐occurrence of AEs when treated with GLP‐1RAs. Finally, we developed a web portal to provide users with the ability to interrogate AEs and GLP‐1RAs from the FAERS data.

From our analysis, we found that AEs associated with GLP‐1RA treatment in weight control/obesity were enriched for metabolism and nutrition disorders, gastrointestinal disorders, and psychiatric disorders. This suggests that individuals taking GLP‐1RAs to control weight should regularly be evaluated for appropriate nutrition intake due to poor diet quality or potential for an eating disorder [15]. In particular, we identified that metabolic acidosis and protein deficiency were AEs enriched in GLP‐1RA usage in weight control/obesity, consistent with several case reports summarized by Fallows [15]. As compared to diabetes usage, weight management/obesity of GLP‐1RAs has been associated with depression disorder and suicidal thoughts, which need to be carefully monitored to prevent potential serious and unwanted outcomes [7]. However, a recent meta‐study comparing GLP‐1RA doses versus placebo without segregating diabetes or weight control/obesity usage found no significant difference in the risk of psychiatric AEs [16]. This suggests that future studies are needed to disentangle the AEs.

GLP‐1RAs used to treat T2D were associated with more serious clinical outcomes, potentially due to the patients being burdened by chronic disease, physically more ill, and worse in their health. Most of the serious AEs were related to the pancreas, such as pancreatic neoplasms. However, recent studies from other real‐world data such as electronic health records (EHRs) showed no significant associations of GLP‐1RAs and cancer risks [17, 18, 19]. Long‐term monitoring and follow‐up may be needed for patients taking GLP‐1RAs to prevent AEs associated with life‐threatening clinical outcomes. In our analysis, we observed high ROR of GLP‐1RAs and retinopathies for diabetes; potentially this could be related to the associated increased risk of nonarteritic anterior ischemic optic neuropathy (NAION) [20]. However, the literature for this association was nuanced; some studies found potential long‐term neurovascular protection but also an increased risk of transient early worsening. This warrants further mechanistic studies and more real‐world data analysis to resolve this association.

FAERS provided real‐world pharmacovigilance in AEs of GLP‐1RAs, yet this database has several limitations and analyses require careful interpretation. Two major limitations of FAERS are underreporting and differential reporting bias in the database. For example, reports of FAERS are often incomplete with various missing data [21]. This could limit associations of certain information in evaluating AEs of drugs, as well as limit clinical details in the database. Although disproportionality methods such as ROR and PRR were used in this study, the results should be interpreted as signals rather than definitive evidence of causality. Therefore, pharmacovigilance should be viewed as hypothesis‐generating and complementary to clinical trial studies.

Future work includes integrating FAERS data with other real‐world data sources such as EHRs, insurance claims, and disease registries to strengthen AE signal validation and contextualize risks in relation to treatment benefits [17, 22]. Advanced computational methods such as machine learning/artificial intelligence approaches in analyzing the real‐world data may further refine detection of biomarkers for GLP‐1RAs AEs, identify phenotypic subgroups at higher risk, and prioritize signals for regulatory monitoring or generating hypotheses.

In conclusion, we identified a wide spectrum of AEs associated with GLP‐1RAs in different treatment contexts (e.g., T2D vs. weight control/obesity). These findings provide a resource for generating hypotheses and shape future research agendas. As millions of patients are taking GLP‐1RAs for weight control and obesity treatment worldwide, clinicians should be vigilant in monitoring for unanticipated long‐term adverse effects. Finally, incorporating real‐world evidence into safety communications and treatment recommendations could reduce unwanted risks in using GLP‐1RAs in disease control and management of obesity.

## Author Contributions

D.S. was responsible for writing the manuscript, implementing the web portal, conducting the analyses, and interpreting the data. M.P. and S.D.H. critically revised the manuscript and contributed to data interpretation. A.C.T. was responsible for writing the manuscript, conducting the analyses, and interpreting the data, provided primary funding, and conceived the overall study design. All authors approved the final version of the manuscript.

## Funding

This research is funded by Dr. Tan's start‐up package from the Huntsman Cancer Institute, University of Utah.

## Conflicts of Interest

The authors declare no conflicts of interest.

## Data Availability

The data that support the findings of this study are available in GLP1RAs and Adverse Events at http://glp1.tanlab.org. These data were derived from the following resources available in the public domain: FDA Adverse Events Reporting System (FAERS), https://www.fda.gov/drugs/fdas‐adverse‐event‐reporting‐system‐faers/fda‐adverse‐event‐reporting‐system‐faers‐latest‐quarterly‐data‐files.
